# Gut microbiota and short-chain fatty acids may be new biomarkers for predicting neonatal necrotizing enterocolitis: A pilot study

**DOI:** 10.3389/fmicb.2022.969656

**Published:** 2022-08-17

**Authors:** Xiao-Chen Liu, Ting-Ting Du, Xiong Gao, Wen-Jing Zhao, Zheng-Li Wang, Yu He, Lei Bao, Lu-Quan Li

**Affiliations:** Neonatal Diagnosis and Treatment Center of Children’s Hospital of Chongqing Medical University, National Clinical Research Center for Child Health and Disorders, Ministry of Education Key Laboratory of Child Development and Disorders, International Science and Technology Cooperation Base of Child Development and Critical Disorders, Chongqing Key Laboratory of Pediatrics, Chongqing, China

**Keywords:** neonatal necrotizing enterocolitis, gut microbiota, short-chain fatty acids, metabolites, predict

## Abstract

**Background:**

Dysbacteriosis is thought to play an important role in the pathogenesis of necrotizing enterocolitis (NEC). We aimed to identify new biomarkers among gut microbiota and short-chain fatty acids (SCFAs) for the early prediction of NEC.

**Materials and methods:**

Thirty-four preterm infants with gestational ages of ≤ 34 weeks who developed gastrointestinal symptoms were divided into the NEC group (*n* = 17) and non-NEC group (*n* = 17). In the NEC group, the gut microbiota and SCFAs in feces were assessed when the infants were enrolled (Group P) and when they were diagnosed with NEC (Group N). In the non-NEC group, samples were assessed when the infants were enrolled (Group C).

**Results:**

The Ace and Chao1 indices were higher in Group P than in Group C (*P* < 0.05), and there was no difference between Groups C and N or between Groups P and N (*P* > 0.05). There was no significant difference in the Simpson and Shannon indices among Groups C, P and N (*P* > 0.05). The four main phyla showed no differences (*P* > 0.05) in composition, while at the genus level, compared with Group C, in Group P, *Clostridioides*, *Blautia* and *Clostridium_sensu_stricto_1* were increased, while *Lactobacillus* and *Bifidobacterium* were decreased (*P* < 0.05). At the species level, *Streptococcus salivarius* and *Rothia mucilaginosa* increased, while *Bifidobacterium animals subsp. lactis* decreased (*P* < 0.05). In Group N, at the genus level, *Stenotrophomonas*, *Streptococcus* and *Prevotella* increased (*P* < 0.05). Compared with those in Group C, the levels of acetic acid, propanoic acid and butyric acid decreased significantly in Groups P and N (*P* < 0.05), and the areas under the curves (AUCs) of these three SCFAs between groups C and P were 0.73, 0.70, and 0.68, respectively.

**Conclusion:**

The increase in *Streptococcus salivarius* and *Rothia mucilaginosa* and decrease in *Bifidobacterium_animals_subsp._lactis*, as well as the decrease in acetic, propionic and butyric acids, may help in the early prediction of NEC.

## Introduction

Neonatal necrotizing enterocolitis (NEC) is one of the most serious gastrointestinal diseases during the neonatal period (Neu and Walker, 2011). In preterm infants, the incidence is 5–12% and up to 13% in those born with very low birth weight. Despite improvements in the management of neonates, the fatality rate is still as high as 20% to 30% and is even higher in those requiring surgery (Foglia et al., 2019; Meister et al., 2020). Severe neurological retardation, enterostenosis after surgery, short bowel syndrome, cholestasis and other complications can subsequently affect the quality of life of infants (Frost et al., 2017; Allendorf et al., 2018). Therefore, there is an urgent need to identify NEC early and intervene as soon as possible.

The diagnosis of NEC depends mainly on clinical features and imaging findings. However, it is always ignored in the early stage, and once the typical features appear, it progresses rapidly in most patients (Neu and Walker, 2011; Meister et al., 2020). Therefore, some biomarkers, such as levels of fecal calprotectin, high mobility group box-1 protein, and intestinal-fatty acid binding protein, have been used to diagnose NEC early (Arboleya et al., 2015; Willers et al., 2020; Huo et al., 2021). However, most of these biomarkers are released due to epithelial cell damage in the intestine with low detection sensitivity in the early stage (Goldstein and Sylvester, 2019).

Epidemiological findings and animal models suggest that dysbacteriosis is one of the risk factors for NEC and plays an important role in the pathogenesis of NEC (Waligora-Dupriet et al., 2005; Schnabl et al., 2008). Previous studies have shown that decreased microbial diversity is one of the features of infants with NEC (Lynch and Pedersen, 2016; Niño et al., 2016; Dobbler et al., 2017; Lee et al., 2020) and that the abundance of *Proteobacteria* is increased while those of *Firmicutes* and *Bacteroidota* are decreased significantly in infants with NEC (Pammi et al., 2017). These symptoms occur 72 h to 7 days before the onset of NEC, so these microbiotas have early predictive value for NEC to some extent (Mai et al., 2011; Tarracchini et al., 2021). In addition, studies have shown that the metabolites of microbiota also play an important role in the disease process and have been regarded as a bridge between microbiota and diseases, including NEC(Macia et al., 2015; Zhu et al., 2016). Therefore, the relationship between microbiota metabolites and NEC has received more attention in recent years (Call et al., 2018; Neu and Pammi, 2018).

Many metabolites, including bile acids, short-chain fatty acids (SCFAs), branched-chain amino acids, trimethylamine N-oxide, tryptophan and indole derivatives, have been reported to be important in physiological functions (McCarville et al., 2020; Agus et al., 2021). Among these metabolites, SCFAs have become a focal point in recent years. SCFAs are organic fatty acids that contain 1–6 carbon atoms and include formic acid, acetic acid, propionic acid, butyric acid, isobutyric acid, valeric acid and isovaleric acid(Koh et al., 2016). Studies have shown that SCFAs play an important role in maintaining the integrity of the intestinal epithelium and repairing the mucosa after injury (Gonçalves et al., 2018; Kayama et al., 2020). For example, propionic acids increase the expression levels of the tight junction proteins zonula occludens-1 and occludin on the epithelial barrier in patients with Parkinson’s disease (Huang et al., 2021), and valeric acid can reduce the incidence of necrotic enteritis in chickens (Onrust et al., 2018). However, few studies have focused on the role of SCFAs in the pathogenesis of NEC, and it remains unclear whether SCFAs are valuable for predicting NEC.

The aim of this study was to explore the value of the gut microbiota and SCFAs in predicting NEC and find new potential biomarkers for the early diagnosis of NEC.

## Subjects and methods

A prospective study enrolling neonates admitted to the Neonatal Diagnosis and Treatment Center of Children’s Hospital of Chongqing Medical University from April to October 2021 was conducted. This study was approved by the Ethics Committee of the Children’s Hospital Affiliated with Chongqing Medical University (No. 2021-32-1) and registered in the China Clinical Trial Center (ChiCTR2100044736). Informed consent forms were obtained from the parents of the enrolled neonates.

### Inclusion and exclusion criteria

The inclusion criteria were as follows: (1) infants with a gestational age less than 34 weeks and (2) infants presenting with one of the following symptoms, namely, abdominal distension, vomiting or bloody stool. The exclusion criteria were as follows: (1) infants who died during hospitalization or were diagnosed with congenital gastrointestinal malformations such as congenital intestinal atresia, megacolon, intestinal malrotation or other gastrointestinal diseases such as food-protein-induced enteritis or lactose intolerance; (2) infants for whom fecal samples were not collected at enrollment or for whom the microbiota and SCFAs were not completely assessed; and (3) infants with a lack of consent to participate in this study.

### Diagnostic criteria

NEC was diagnosed based on Bell’s diagnostic criteria (Kliegman and Walsh, 1987). Infants who met the following criteria were enrolled in the NEC group: (1) one or more of the systemic symptoms, including drowsiness, an unstable body temperature, apnea, and bradycardia and/or (2) had one or more of the clinical signs, including gastric aspirate with bile or emesis, abdominal distention, and occult and/or gross bloody stool, and (3) had at least one of the imaging findings, including pneumatosis intestinalis, portal vein gas and/or pneumoperitoneum. The non-NEC infants enrolled at the same time were matched 1:1 according to gestational age and birth weight. The gestational age difference was less than 1 week, and the birth weight difference was less than 250 grams. Feeding intolerance (FI) in the non-NEC group was diagnosed based on the following criteria: (1) gastric retention exceeding 50% of the previous feeding volume; (2) the presence of emesis, abdominal distention or both; and (3) a failed enteral feeding plan including a decrease, detention, or discontinuity.

### Data collection

Relevant clinical data of the enrolled infants were collected, including baseline information, risk factors for prenatal (maternal, gestational and intrapartum) and pre-enrollment (feeding, antibiotic use, invasive procedures, etc.) conditions, and hospitalization outcomes (duration, surgery and other complications).

### Faecal sampling and grouping progress

**FIGURE 1:**
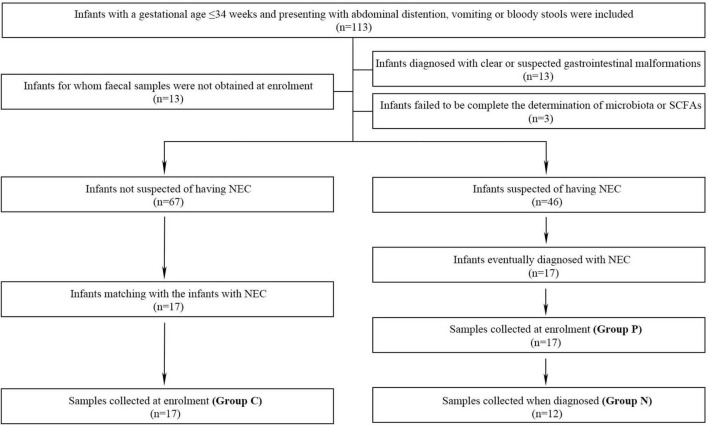
The inclusion, exclusion and grouping processes of the study.

Naturally-excreted fecal samples were collected with disposable sterile swabs. For infants who ultimately developed NEC, fecal samples were collected at two different time-points to identify the changes in the microbiota and SCFAs with the development of disease. The two time-points were when the infants were enrolled (Group P) and when they were prospectively diagnosed with NEC (Group N). For infants who did not develop NEC, samples were only collected when they were enrolled (Group C) (Figure 1). Fresh samples were immediately transferred to the laboratory, and every 250 mg was aliquoted into 1.5-ml sterile enzymatic EP tubes, which were then stored in a freezer at −80°C.

### Microbiota determination

Genomic DNA of the fecal microbiota was extracted with a QIAamp FAST DNA Stool Mini-Kit (Qiagen, Hilden Germany) as previously described (Liu et al., 2022). DNA extracts were tested on a 1% agarose gel, and the concentration and purity were assessed with a spectrophotometer (NanoDrop 2000 UV–vis, Thermo Scientific, Waltham, MA, USA). The V3-V4 hypervariable region of the 16S rDNA genes was amplified with primers 338F (5′-ACTCCTACGGG AGGCAGCAG-3′) and 806R (5′-GGACTACHVGGGTWTCTAAT-3′). The cycles were as follows: (1) initial denaturation at 95°C for 3 min; (2) denaturation at 95°C for 30 seconds; (3) annealing at 55°C for 30 seconds; and (4) extension at 72°C for 45 seconds. After 27 cycles, an extension at 72°C for 10 min was performed. The products were extracted and recovered by 2% agarose gel electrophoresis (Axygen Biosciences, United States) and then quantified using a Quantus Fluorometer (Promega, United States). Finally, amplicons were pooled in equimolar amounts and subjected to paired-end sequencing (2 × 250) on the Illumina MiSeq platform (San Diego, CA, United States) following standard protocols. Reads were differentiated based on primers and barcodes, and sequence orientation was adjusted to ensure accurate barcode matching.

Raw microbiota data were processed with QIIME (version 1.9.1; Boulder, CO, United States). Briefly, bases with a quality score less than 20 were truncated, and sequences longer than 10 bp overlapped. Reads in overlapping regions of the spliced sequences that exceeded the maximum mismatch ratio of 0.2 were deleted. Sequences were ultimately divided into operational taxonomic units (OTUs) using UPARSE (version 7.0.1090; La Jolla, CA, United States), and OTUs were clustered with a similarity threshold of 97% (Amato et al., 2013). Species classification was performed using silva138/16s_bacteria taxonomic data with a classification confidence of 70%.

### Short-chain fatty acids (SCFAs) measurement

The methods have been previously described (Liu et al., 2022). Standards for acetic, propionic, butyric, isobutyric, valeric, isovaleric, and hexanoic acids were prepared with ethyl acetate and 4-methylvaleric acid to standard concentration gradients of 0.1 μg/mL, 0.5 μg/mL, 1 μg/mL, 5 μg/mL, 10 μg/mL, 20 μg/mL, 50 μg/mL and 100 μg/mL. Thirty milligrams of each fecal sample was thawed, and 900 μL of 0.5% phosphoric acid was added to resuspend the sample, which was then shaken for 2 min and centrifuged at 14,000 × *g* for 10 min. Then, 800 μL of the supernatant was added to an equal amount of ethyl acetate to repeat the above processes, and 600 μL of the upper organic phase was added to 4-methylvaleric acid as an internal standard. The samples were separated by a DB-WAX capillary column (30 m × 0.25 mm ID × 0.25 μm) and analyzed by 7890A/5975C gas chromatography–mass spectrometry (Agilent, Santa Clara, CA, United States). Finally, MSD ChemStation software (Agilent, Santa Clara, CA, United States) was used to extract chromatographic peak areas and retention times. Standard curves were drawn, and the SCFAs content was calculated.

### Data analysis

All the clinical data, alpha diversity index values and SCFAs contents were analyzed with SPSS statistical software (version 24; Chicago, IL, United States). Normally distributed data are presented as the mean ± standard deviation (SD) and were compared with *matched samples t tests* between two groups. Non-normally distributed measurement data are presented as medians (interquartile ranges, IQRs), and the *Wilcoxon signed rank-sum test* was used. The comparison of SCFAs at different time-points in one infant was performed with *a generalized linear mixed model*. Count data were analyzed by *Fisher’s exact test*. The comparisons of the gut microbiota composition were performed with the *Kruskal–Wallis H test*. Principal coordinate analysis (PCoA) based on the Euclidean distance matrix of the beta diversity and other statistics was performed with R language (version 3.3.1; Auckland, New Zealand). Correlation heatmap analysis was conducted to show the relationship between SCFAs concentrations and the microbiota composition based on *Spearman rank correlation* in R. Receiver operating characteristic (ROC) curve analysis was performed with GraphPad Prism (version 9.0; La Jolla, CA, United States). *P* < 0.05 was regarded as statistically significant. Figures illustrating the microbiota results were drafted with R language or GraphPad Prism.

## Results

### Clinical information

One hundred thirteen preterm infants admitted to our department were enrolled, and 46 infants were suspected to have NEC at the time of enrollment. Among them, 29 infants were excluded from further study due to obvious or suspected gastrointestinal malformations (*n* = 13), the failure to collect fecal samples at the time of enrollment (*n* = 13) and incomplete determination of the microbiota and SCFAs (*n* = 3). Therefore, 17 infants with NEC were enrolled, and 5 developed intestinal perforation on the day of enrollment (Figure 1). Compared with the non-NEC group, the NEC group had a larger corrected gestational age at the time of enrollment. Those with NEC had a higher incidence of septicemia and longer hospitalization stays (*P* < 0.05). There were no significant differences in other factors, including general information, prenatal risk factors and other risk factors, before enrollment (*P* > 0.05) (Table 1).

**TABLE 1:** Clinical features of the infants enrolled in this study.

	Non-NEC (*n* = 17)	NEC (*n* = 17)	*X ^2^*/*Z*/*t*	*P*
**General information**				
Male, % (n)	52.9 (9)	76.5 (13)	/	0.141
Admission age, M (IQR), h	0.08 (0.04,2.54)	0.08 (0.06,3.38)	−0.489	0.624
Gestational age, $\bar{\text{x}}$ ± S. D, w	30.48 ± 1.943	30.50 ± 2.120	−0.081	0.936
Birth weight, $\bar{\text{x}}$ ± S. D, g	1442.35 ± 346.433	1427.35 ± 459.763	0.183	0.857
Cesarean section, % (n)	41.2 (7)	35.3 (6)	/	0.500
Apgar 1 min, M (IQR)	8.00 (6.00,9.00)	8.00 (7.00,9.00)	−0.996	0.319
Apgar 5 min, M (IQR)	9.00 (8.50,10.00)	9.00 (9.00,9.50)	−0.965	0.334
**Prenatal risk factors**				
PROM, % (n)	23.5 (4)	29.4 (5)	/	0.500
Intrauterine distress, % (n)	11.8 (2)	5.9 (1)	/	0.500
Maternal hypertension, % (n)	11.8 (2)	5.9 (1)	/	0.500
GDM, % (n)	11.8 (2)	23.5 (4)	/	0.328
Chorioamnionitis, % (n)	11.8 (2)	5.9 (1)	/	0.500
Antenatal steroid use, % (n)	82.4 (14)	64.7 (11)	/	0.219
Antenatal antibiotic use, % (n)	23.5 (4)	0.0 (0)	/	0.051
**Risk factors before enrollment**				
Formula milk feeding, % (n)	70.6 (12)	58.8 (10)	/	0.360
Antibiotic course, M (IQR), d	5.00 (2.50,7.50)	4.00 (3.00,10.00)	−0.259	0.796
Endotracheal intubation, % (n)	5.9 (1)	5.9 (1)	/	0.758
PICC period, $\bar{\text{x}}$ ± S. D, d	12.00 ± 11.040	19.18 ± 18.762	−1.737	0.102
PN courses, $\bar{\text{x}}$ ± S. D, d	13.18 ± 11.690	18.29 ± 15.206	−1.384	0.186
Transfusion, % (n)	17.6 (3)	35.3 (6)	/	0.219
CGA when enrolling, $\bar{\text{x}}$ ± S. D, w	32.52 ± 1.794	33.99 ± 2.385	−3.763	0.002
Onset age of NEC, $\bar{\text{x}}$ ± S. D, d	/	30.24 ± 15.888	/	/
Days from enrollment to the onset of NEC, $\bar{\text{x}}$ ± S. D, d	/	7.00 ± 7.640	/	/
**Outcome**				
Surgery, % (n)	0.0 (0)	23.5 (5)	/	0.051
Septicemia, % (n)	23.5 (4)	88.2 (15)	/	0.000
IVH, % (n)	17.6 (3)	23.5 (4)	/	0.500
BPD,% (n)	47.1 (8)	41.2 (7)	/	0.500
Shock, % (n)	5.9 (1)	23.5 (4)	/	0.168
Hospital stays, $\bar{\text{x}}$ ± S. D, d	48.41 ± 26.003	83.35 ± 47.911	−3.111	0.007

NEC, necrotizing enterocolitis; PROM, premature rupture of membranes > 18 h; GDM, Gestational diabetes mellitus; PICC, peripherally inserted central catheter; PN, Parenteral nutrition; CGA, Corrected gestational age; IVH, Intraventricular hemorrhage; BPD, Bronchopulmonary dysplasia.

### Microbiota characteristics

Determination and analysis of the microbiota were performed for 46 samples, with 17 from Group P and 5 from infants diagnosed on the enrollment day with intestinal perfusion; only 12 samples were assessed for Group N. According to the pairing principle, 17 samples in Group C were obtained (Figure 1). *Core* analysis was performed to verify whether the sample size was sufficient, and the curve eventually flattened, which means that the sample size was reasonable (Supplementary Figure 1A). Analysis of similarities (ANOSIM) based on the Bray–Curtis distance algorithm showed that the difference among the three groups was not significantly greater than that within the groups, indicating that the grouping was comparable (Supplementary Figure 1B). A rarefaction curve was drawn based on the Shannon diversity index, and the flat curves showed that the number of sequences measured was sufficient to reflect the vast majority of microbial diversity information (Supplementary Figure 1C).

### Diversity analysis

**FIGURE 2:**
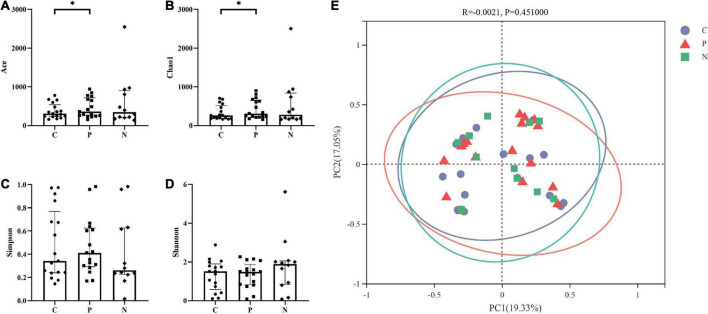
Alpha and beta diversity among Groups C, P, and N. The Ace and Chao1 indices in Group C were higher than those in Group P (*P* < 0.05), but there were no differences between Groups C and N or between Groups P and N (*P* > 0.05). **(A,B)** The Simpson and Shannon indices showed no difference among Groups C, P and N (*P* > 0.05). **(C,D)** There was no significant difference in the beta diversity among the three groups (*P* > 0.05) **(E)**.

Alpha diversity was assessed based on the Ace and Chao1 richness indices and the Simpson and Shannon diversity indices. The Ace and Chao1 indices in Group C were higher than those in Group P (*P* < 0.05), and the Simpson and Shannon indices showed no difference (*P* > 0.05). Groups C and N showed no differences in the four indices. In addition, there were no differences between Groups P and N (*P* > 0.05) (Figures 2A–D).

PCoA showed that the samples in Groups C, P and N were all relatively discrete, and the explanatory values on the X and Y axes were 19.33% and 17.05%, respectively. ANOSIM showed that there was no significant difference in the beta diversity among the three groups (*P* > 0.05) (Figure 2E).

### Composition of microbiota

The number of OTUs in the three groups and the shared relationship are shown in Venn diagrams. At the phylum level, *Proteobacteria*, *Firmicutes*, *Actinobacteriota* and *Bacteroidota* were the dominant phyla, and on the ternary phase diagram, the phyla with a total amount of less than 1% were combined, which means that the four main phyla accounted for more than 99% of the total composition. At the genus level, the similarities and differences among the three groups are shown in the heatmap, and different colors show the abundance of different genera. *Enterococcus*, *Escherichia-Shigella*, *Staphylococcus*, *Enterobacter*, *Klebsiella*, and *Acinetobacter* were the main genera, and their relationships with the phyla are presented in the phylogenetic tree (Supplementary Figure 2).

**FIGURE 3:**
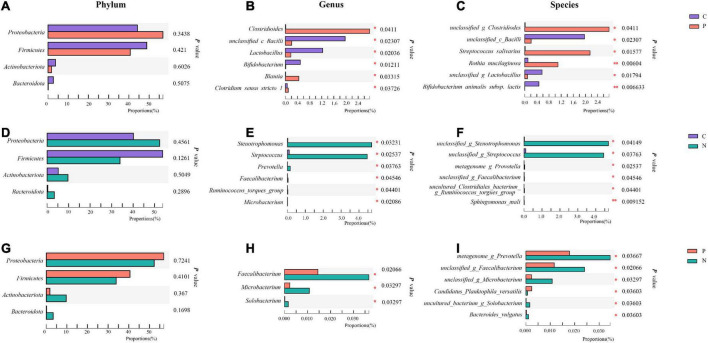
Community abundance of the gut microbiota in Groups C, P, and N. Differences between Groups C and P at the phylum **(A)**, genus **(B)** and species **(C)** levels. Differences between Groups C and N at the phylum **(D)**, genus **(E)** and species **(F)** levels. Differences between Groups P and N at the phylum **(G)**, genus **(H)** and species **(I)** levels. *P < 0.05, ^**^P < 0.01.

When compared with Group C, in Group P, *Proteobacteria* increased while *Firmicutes, Actinobacteriota* and *Bacteroidota* decreased at the phylum level, but no differences were observed (*P* > 0.05). *Clostridioides, Blautia* and *Clostridium_sensu_stricto_1* increased, while *unclassified_c_Bacilli, Lactobacillus* and *Bifidobacterium* decreased significantly at the genus level (*P* < 0.05). At the species level, *unclassified_g_Clostridioides, Streptococcus salivarius* and *Rothia mucilaginosa* increased, while *unclassified_c_Bacilli, unclassified_g_Lactobacillus* and *Bifidobacterium animals subsp. lactis* decreased (*P* < 0.05) (Figures 3A–C).

When Groups C and N were compared, in Group N, only *Firmicutes* decreased, while the other three main phyla increased at the phylum level without significant differences (*P* > 0.05). *Stenotrophomonas, Streptococcus* and *Prevotella* increased at the genus level. At the species level, *unclassified_g_Stenotrophomonas* and *unclassified_g_Streptococcus* increased (*P* < 0.05) (Figures 3D–F).

When Groups P and N were compared, in Group N, *Proteobacteria* and *Firmicutes* decreased, while *Actinobacteriota* and *Bacteroidota* increased without a significant difference (*P* > 0.05). At the genus level, *Faecalibacterium, Microbacterium* and *Solobacterium* increased, and at the species level, *metagenome_g_Prevotella, unclassified_g_Faecalibacterium, unclassified_g_Microbacterium, uncultured_bacterium_g_ Solobacterium* and *Bacteroides_vulgatus* increased, while *Candidatus_Planktophila_versatilis* decreased (*P* < 0.05). However, these genera and species accounted for a very small proportion of the total (Figure 3G–I).

### Short-chain fatty acids (SCFAs) measurement

**FIGURE 4:**
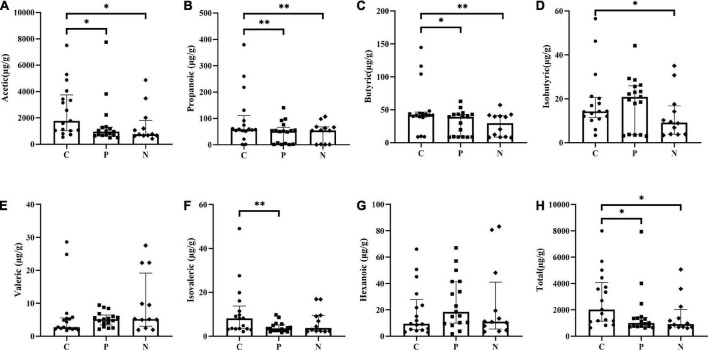
Comparison of short-chain fatty acids (SCFAs) concentrations among Groups C, P and N. **(A)** Acetic acid, **(B)** propanoic acid, **(C)** butyric acid, **(D)** isobutyric acid, **(E)** valeric acid, **(F)** isovaleric acid, **(G)** hexanoic acid, and **(H)** total SCFAs. *P < 0.05, ^**^P < 0.01.

**FIGURE 5:**
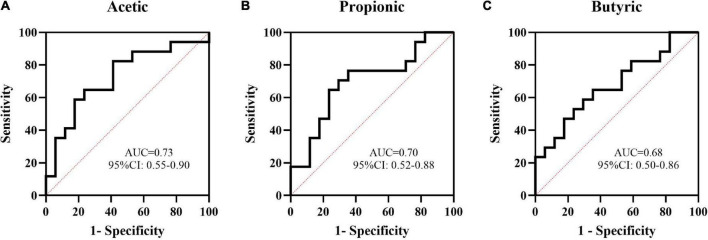
The value of some short-chain fatty acids (SCFAs) in the prediction of NEC by ROC curve analysis. The AUCs of acetic **(A)**, propanoic **(B),** and butyric **(C)** acids between Groups C and P were 0.73, 0.70, and 0.68, respectively.

Acetic, propanoic, butyric and isovaleric acids decreased significantly in Group P compared with Group C, and acetic, propanoic, butyric and isobutyric acids decreased in Group N compared with Group C. Additionally, the total amount of SCFAs in Groups P and N was lower than that in Group C (*P* < 0.05). Between Groups P and N, there were no differences in any of the SCFAs (*P* > 0.05) (Figure 4). To determine the value of SCFAs in predicting NEC, ROC curves of acetic, propanoic and butyric acids between Groups C and P were generated, and the AUCs were 0.73, 0.70, and 0.68, respectively (Figure 5).

### Relationship between short-chain fatty acids (SCFAs) and the gut microbiota

**FIGURE 6:**
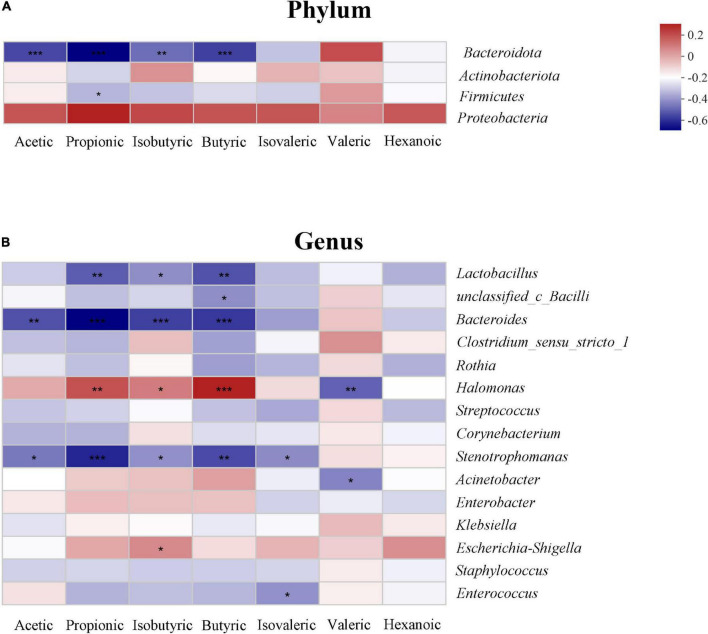
Relationship between the gut microbiota and short-chain fatty acids (SCFAs) on phylum **(A)** and genus level **(B)** in the study. The change in color reflects the data in the two-dimensional matrix. The color depth indicates the size of the value, and it can intuitively express the size of the value in a defined color depth.

The relationship between SCFAs and the gut microbiota is shown in the heatmap. At the phylum level, acetic, propanoic, butyric and isobutyric acids were negatively correlated with *Bacteroidota*, and propanoic acid was positively correlated with *Firmicutes* (*P* < 0.05). All SCFAs were positively correlated with *Proteobacteria*; however, there was no statistically significant difference (*P* > 0.05) (Figure 6A). At the genus level, propanoic, butyric and isobutyric acids were positively correlated with *Halomonas*, and most SCFAs were negatively correlated with *Lactobacillus*, *Bacteroides*, and *Stenotrophomonas* (*P* < 0.05) (Figure 6B).

## Discussion

New biomarkers for the early prediction of the onset of NEC are important. In this study, we found that *Streptococcus salivarius* and *Rothia mucilaginosa* increased and *Bifidobacterium subsp. lactis* decreased 7 days before NEC occurred and that acetic, propionic and butyric acid decreased, which showed great value for the early prediction of NEC.

In our study, *Proteobacteria* increased, while *Firmicutes*, *Actinobacteriota* and *Bacteroidota* decreased at the phylum level, although without significance, and at the genus level, *Clostridioides, Blautia* and *Clostridium_sensu_stricto_1* increased, while *unclassified_c_Bacilli, Lactobacillus* and *Bifidobacterium* increased in infants with NEC. Consistent with our study, *Clostridium* and *Bacillus* have been previously considered as biomarkers for predicting NEC (de la Cochetiere et al., 2004; Olm et al., 2019; Brehin et al., 2020). However, no studies have clearly proposed which bacteria are closely related to NEC. Therefore, we further analyzed the relevant microbiota at the species level and found that *Streptococcus salivarius* and *Rothia mucilaginosa* increased and *Bifidobacterium subsp. lactis* decreased in the early stage of NEC. In addition, although we found that bacterial species can help to predict NEC, the prediction is limited by the detection methods; some bacteria are identified as unclassified or uncultured species, and some anaerobic bacteria cannot be easily detected clinically.

Our study found that SCFAs may be better biomarkers that can reflect the overall characteristics of the microbiota and can be determined more easily. We found that acetic, propionic and butyric acid levels decreased significantly, and that ROC curve analysis showed predictive values before the diagnosis of NEC. Combined with the heatmap, the production of metabolites was related to the decline in *Firmicutes* and *Bacteroidota*, although there was no difference in their abundance at the phylum level. It might be the joint work of the gut microbiota to produce metabolites.

Acetic, propionic and butyric acids are the main SCFAs, which account for approximately 90-95% of the total SCFAs in the human intestines (Soldavini and Kaunitz, 2013) and are mainly produced by the fermentation of dietary fiber by microbiota (McNabney and Henagan, 2017; Ghonimy et al., 2018). The production of acetic acids is distributed across different genera, such as *Bacteroides*, *Bifidobacterium*, *Lactobacillus* and *Prevotella*, which ferment pyruvate in the acetyl-CoA or Wood-Ljungdahl pathway. Propionic acid is mainly produced from succinate by *Bacteroides* and *Veillonella* via the succinate pathway or from lactate by *Propionibacterium* via the acrylate pathway or propylene glycol pathway. Butyric acid is mainly produced by *Clostridium*, *Lactobacillus*, and *Clostridium perfringens* from acetyl-CoA and butyryl-CoA via the typical pathway or from lactate and acetate via the lactate pathway (den Besten et al., 2013). Previous studies have shown that SCFAs play important roles in intestinal inflammation and are involved in intestinal injury. Li et al. found that a certain concentration of acetic acid alleviated high-carbohydrate-induced intestinal inflammation by inhibiting MAPK activation and NF-κB phosphorylation (Li et al., 2020). Pace et al. showed that propionic and butyric acids can inhibit the intestinal inflammatory response in a human model by reducing the expression of the proinflammatory cytokines Mcp-1 and IL-8 and the gene transcription of chemotactic cytokine family members (Pace et al., 2021). Our previous study found that butyrate intervention attenuated intestinal inflammation and partially corrected dysbacteriosis in mice with NEC (Sun et al., 2021). Thus, the changes in acetic, propionic and butyric acids in our study can reflect the gut conditions caused by NEC and may be helpful for early prediction. In addition, the detection method is simple, and the detection duration is greatly shortened compared with that for detecting the gut microbiota, which makes it easier to be applied clinically.

Walker and Claud first proposed the hypothesis that decreased diversity may lead to NEC in 2001 (Claud and Walker, 2001), which was confirmed by previous studies. However, in our study, the richness of the gut microbiota in the NEC group before the onset of NEC was higher than that in the control group, and the diversity indices and beta diversity showed no differences among the three groups. The larger corrected gestational age in infants with NEC might be the cause of increased richness, which has a strong influence on the development of the gut microbiota during the neonatal period, especially in preterm infants (La Rosa et al., 2014). The infants enrolled in our study were those who presented with gastrointestinal symptoms, and those in Group C were mostly diagnosed with FI. FI can lead to a decrease in diversity and changes in beta diversity, and studies have shown a pattern similar to NEC in patients who developed FI (La Rosa et al., 2014; Li et al., 2021; Liu et al., 2022), which can explain why the diversity showed no differences in our study.

There were some limitations to our study. Our prospective study enrolled infants with gastrointestinal symptoms, but the difference in the time to disease development was ignored, which may affect the microbiota. Second, for the small sample size, the diversity of the microbiota showed no difference, and the predictive value of SCFAs was low in the ROC curve analysis. Further studies with larger samples are needed to make our conclusion more convincing.

## Conclusion

*Streptococcus salivarius* and *Rothia mucilaginosa* increased, *Bifidobacterium_animals_subsp._lactis* decreased and acetic, propionic and butyric acids decreased before the diagnosis of NEC, which may help in the early prediction of NEC.

## Data availability statement

The datasets for this study can be found in the NCBI (BioProject ID: PRJNA832593 http://www.ncbi.nlm.nih.gov/bioproject/832593) and obtained from the corresponding author.

## Ethics statement

The studies involving human participants were reviewed and approved by Ethics Committee of the Children’s Hospital Affiliated with Chongqing Medical University. Written informed consent to participate in this study was provided by the participants’ legal guardian/next of kin.

## Author contributions

All the authors made substantial contributions to the study and manuscript and meet the criteria for authorship defined in the author instructions. X-CL and T-TD collected the clinical data and worked on basic sample processing and the drafting of the manuscript. X-CL, T-TD, XG, and W-JZ collected the fecal samples. Z-LW, YH, and L-QL supervised the project and contributed to the conception and design of the study and analysis and interpretation of the data. L-QL contributed to the critical revision and final approval of the manuscript. X-QL, T-TD, and L-QL performed the final approval of the manuscript. All authors contributed to the article and approved the submitted version.

## Supplementary material

Supplementary Figure 1 Verification of the sample size, grouping and sequences. *Core* analysis showed that the curve eventually flattened, and the sample size was reasonable. **(A)** Analysis of similarities (ANOSIM) showed that the difference among the three groups was not significantly greater than that within the groups, which means that the three groups were comparable. **(B)** The flat rarefaction curve showed that the numbers of sequences measured were enough to reflect the diversity information **(C)**.

Supplementary Figure 2 Composition of the microbiota on different levels. Venn diagrams showing the numbers and shared relationships of OTUs in Groups C, P and N. **(A)** The phylum level was dominated by *Proteobacteria*, *Firmicutes*, *Actinobacteriota* and *Bacteroidota.* **(B)**, and the main phyla accounted for more than 99% of the total composition **(E)**. The similarities and differences among the three groups at the genus level and the abundance of different genera are shown in the heatmap. **(F)** *Enterococcus*, *Escherichia-Shigella*, *Staphylococcus*, *Enterobacter*, *Klebsiella*, and *Acinetobacter* were the main genera, and their relationships with the phyla are presented in the phylogenetic tree **(E)**.
