# Supplementary material for: Gut microbiota and short-chain fatty acids may be new biomarkers for predicting neonatal necrotizing enterocolitis: A pilot study
Source: Front Microbiol. 2022 Aug 17;13:969656. doi: 10.3389/fmicb.2022.969656 (PMC9428482; doi:10.3389/fmicb.2022.969656)
Supplement: Supplementary Figure 1 — Verification of the sample size, grouping and sequences. Core analysis showed that the curve eventually flattened, and the sample size was reasonable. (A) Analysis of similarities (ANOSIM) showed that the difference among the three groups was not significantly greater than that within the groups, which means that the three groups were comparable. (B) The flat rarefaction curve showed that the numbers of sequences measured were enough to reflect the diversity information (C). [file Data_Sheet_1.docx]

Supplementary Material


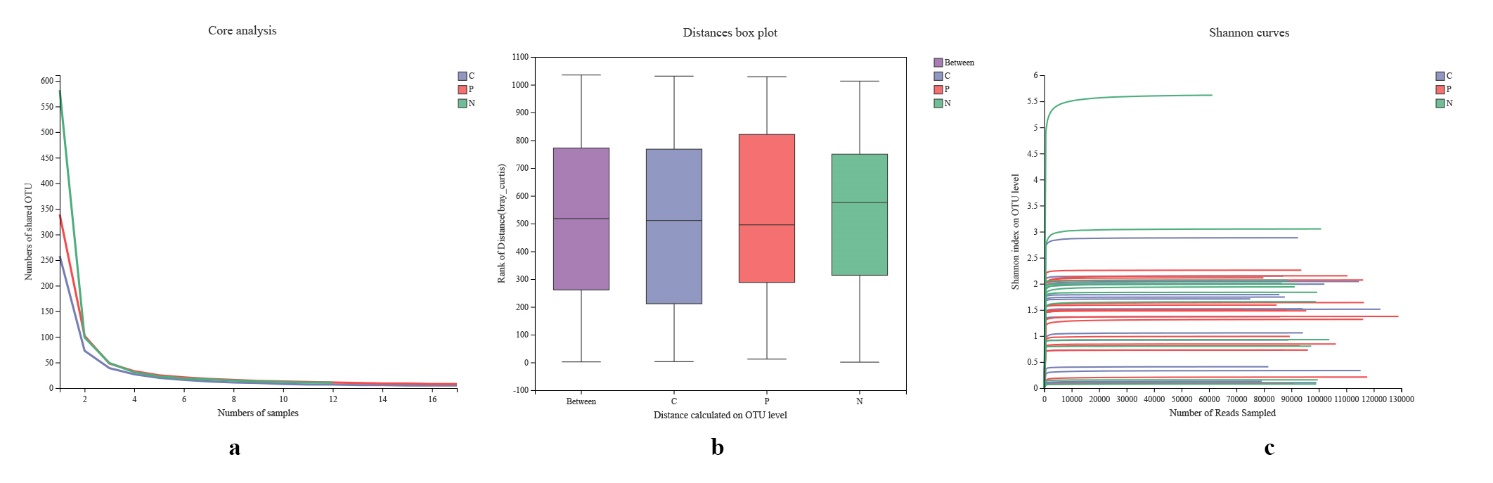


**Figure S1 Verification of the sample size, grouping and sequences.** *Core* analysis showed that the curve eventually flattened, and the sample size was reasonable. **(a)** Analysis of similarities (ANOSIM) showed that the difference among the three groups was not significantly greater than that within the groups, which means that the three groups were comparable. **(b)** The flat rarefaction curve showed that the numbers of sequences measured were enough to reflect the diversity information. **(c)**


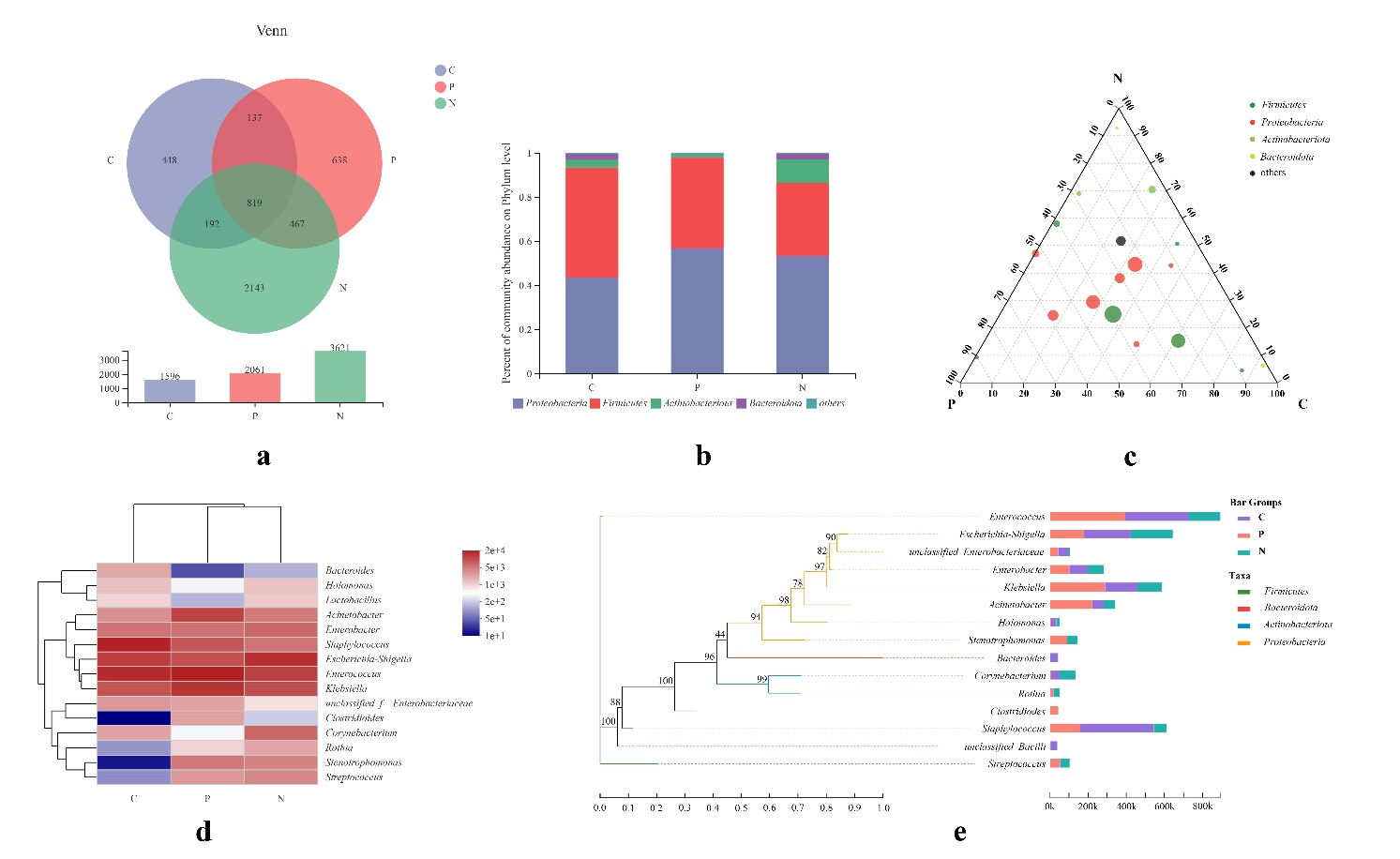


**Figure S2 Composition of the microbiota on different levels.** Venn diagrams showing the numbers and shared relationships of OTUs in Groups C, P and N. **(a)** The phylum level was dominated by *Proteobacteria*, *Firmicutes*, *Actinobacteriota* and *Bacteroidota.* **(b)**, and the main phyla accounted for more than 99% of the total composition **(e)**. The similarities and differences among the three groups at the genus level and the abundance of different genera are shown in the heatmap. **(f)** *Enterococcus*, *Escherichia-Shigella*, *Staphylococcus*, *Enterobacter*, *Klebsiella*, and *Acinetobacter* were the main genera, and their relationships with the phyla are presented in the phylogenetic tree. **(e)**
